# Streptococcus pyogenes Φ1207.3 Is a Temperate Bacteriophage Carrying the Macrolide Resistance Gene Pair *mef*(A)-*msr*(D) and Capable of Lysogenizing Different Streptococci

**DOI:** 10.1128/spectrum.04211-22

**Published:** 2023-01-10

**Authors:** Francesco Santoro, Gabiria Pastore, Valeria Fox, Marie-Agnes Petit, Francesco Iannelli, Gianni Pozzi

**Affiliations:** a Laboratory of Molecular Microbiology and Biotechnology, Department of Medical Biotechnologies, University of Siena, Siena, Italy; b Université Paris-Saclay, INRAE, AgroParisTech, Micalis, Jouy-en-Josas, France; Università Roma Tre

**Keywords:** temperate phage, lysogenization, *Streptococcus pyogenes*, *Streptococcus pneumoniae*, Φ1207.3, mitomycin C, transmission electron microscopy, qPCR, phi1207.3

## Abstract

Streptococcus pyogenes prophage Φ1207.3 (formerly Tn*1207.3*) carries the *mef*(A)-*msr*(D) resistance genes, responsible for type M macrolide resistance. To investigate if Φ1207.3 is a functional bacteriophage, we transferred the element from the original S. pyogenes host in a prophage-free and competence-deficient S. pneumoniae strain. Pneumococcal cultures of the Φ1207.3-carrying lysogen were treated with mitomycin C to assess if Φ1207.3 enters the lytic cycle. Mitomycin C induced a limited phage burst and a growth impairment, resulting in early entrance into the stationary phase. To determine if Φ1207.3 is able to produce mature phage particles, we prepared concentrated supernatants recovered from a mitomycin C-induced pneumococcal culture by sequential centrifugation and ultracentrifugation steps. Negative-staining transmission electron microscopy (TEM) of supernatants revealed the presence of phage particles with an icosahedral, electron-dense capsid and a long, noncontractile tail, typical of a siphovirus. Quantification of Φ1207.3 was performed by quantitative PCR (qPCR) and semiquantitatively by TEM. PCR quantified 3.34 × 10^4^ and 6.06 × 10^4^ excised forms of phage genome per milliliter of supernatant obtained from the untreated and mitomycin C-treated cultures, respectively. By TEM, we estimated 3.02 × 10^3^ and 7.68 × 10^3^ phage particles per milliliter of supernatant. The phage preparations of Φ1207.3 infected and lysogenized pneumococcal recipient strains at a frequency of 7.5 × 10^−6^ lysogens/recipient but did not show sufficient lytic activity to form plaques. Phage lysogenization efficiently occurred after 30 min of contact of the phages with the recipient cells and required a minimum of 10^3^ phage particles.

**IMPORTANCE** Bacteriophages play an important role in bacterial physiology and genome evolution. The widespread use of genome sequencing revealed that bacterial genomes can contain several different integrated temperate bacteriophages, which can constitute up to 20% of the genome. Most of these bacteriophages are only predicted *in silico* and are never shown to be functional. In fact, it is often difficult to induce the lytic cycle of temperate bacteriophages. In this work, we show that Φ1207.3, a peculiar bacteriophage originally from Streptococcus pyogenes, which can lysogenize different streptococci and carries the macrolide resistance *mef*(A)-*msr*(D) gene pair, is capable of producing mature virions, but only at a low level, while not being able to produce plaques. This temperate phage is probably a partially functional phage, which seems to have lost lytic characteristics to specialize in lysogenization. While we are not used to conceiving phages separately from lysis, this behavior could actually be more frequent than expected.

## INTRODUCTION

Mobile genetic elements (MGEs) are segments of DNA that encode enzymes and proteins, mediating their movement. All the MGEs are globally referred to as the mobilome (1) and represent a primary source of diversity for prokaryotes since they can have profound effects on bacterial genome evolution by different means, such as by broadening the gene repertoire or by disrupting existing genes (2, 3). These elements are also involved in the capture and spread of antimicrobial resistance genes. The MGEs usually implicated in the dissemination of antibiotic resistance genes are plasmids and integrative conjugative elements (ICEs) (4). Bacteriophages are the most abundant entities in the biosphere, with an estimated total number of virus-like particles on Earth close to 10^31^ (5–8). Nonetheless, phages are rarely found to carry antibiotic resistance genes (9), and their transfer rarely occurs between different bacterial species. We previously described a 52,491-bp genetic element integrated at a specific site into the chromosome of the Streptococcus pyogenes clinical strain 2812A (10, 11). Since the element was transferred to other streptococcal species with a transfer mechanism resembling conjugation, it was named Tn*1207.3*. DNA sequence analysis showed that, besides the *mef*(A)-*msr*(D) macrolide efflux gene pair (12–14), the element contains phage genes and thus was renamed Φ1207.3. In S. pyogenes clinical isolates, other *mef*(A)-*msr*(D)-carrying prophages were found, including Φ10394.4, Φ29862, Φ29961, Φ29854, and Φm46.1 and its variant, VP_00501.1 (15–19). To determine whether a prophage is fully functional, several properties of the element need to be checked. It should first be able to excise its genome, then produce mature virions, and, finally, infect new hosts and make plaques, or, at least, lysogenize its host. To investigate if Φ1207.3 is a fully functional bacteriophage, we transferred it into an S. pneumoniae prophage-free competence-deficient laboratory strain where we demonstrated that Φ1207.3 is a bacteriophage able to excise, produce mature phage particles with a siphovirus morphology, and lysogenize a sensitive recipient.

## RESULTS

### Transfer of Φ1207.3 to Streptococcus pneumoniae.

To investigate if Φ1207.3 is a functional bacteriophage, the genetic element was transferred from the original clinical Streptococcus pyogenes host in a prophage-free, competence-deficient pneumococcal strain. The resulting pneumococcal FR1 lysogen strain was obtained with a mating experiment where S. pyogenes 2812A was the Φ1207.3 donor and S. pneumoniae FP10 was the recipient (Table 1). Transfer of Φ1207.3 occurred at a frequency of 3.8 × 10^−5^ ± 7.6 × 10^−6^ lysogens per donor, as already observed (20).

**TABLE 1:** Bacterial strains and relevant properties^*a*^

Strain	Properties	Reference(s) or source
2812A	Streptococcus pyogenes, Italian clinical strain, serotype M1, carrying Φ1207.3, Em^r^	10
Rx1	Streptococcus pneumoniae, rough highly transformable laboratory strain, *comC1-comD1*, Hex^−^	40
FP10	Rx1 derivative, competence deficient (Δ*comC1*), *str-41*, Cm^r^, Sm^r^	20, 34, 41
FP11	Rx1 derivative, competence deficient (Δ*comC1*), *nov-1*, Cm^r^, Nov^r^	20, 34, 41
FR1	FP10 derivative carrying Φ1207.3, Cm^r^, Sm^r^, Em^r^	This study

a Em, erythromycin; Cm, chloramphenicol; Nov, novobiocin; Sm, streptomycin; *comC1-comD1* encode the competence stimulating peptide and its receptor; Hex is the DNA mismatch repair system; the *str-41* allele confers streptomycin resistance; the *nov-1* allele confers novobiocin resistance.

### Φ1207.3 induction by mitomycin C.

**FIG 1:**
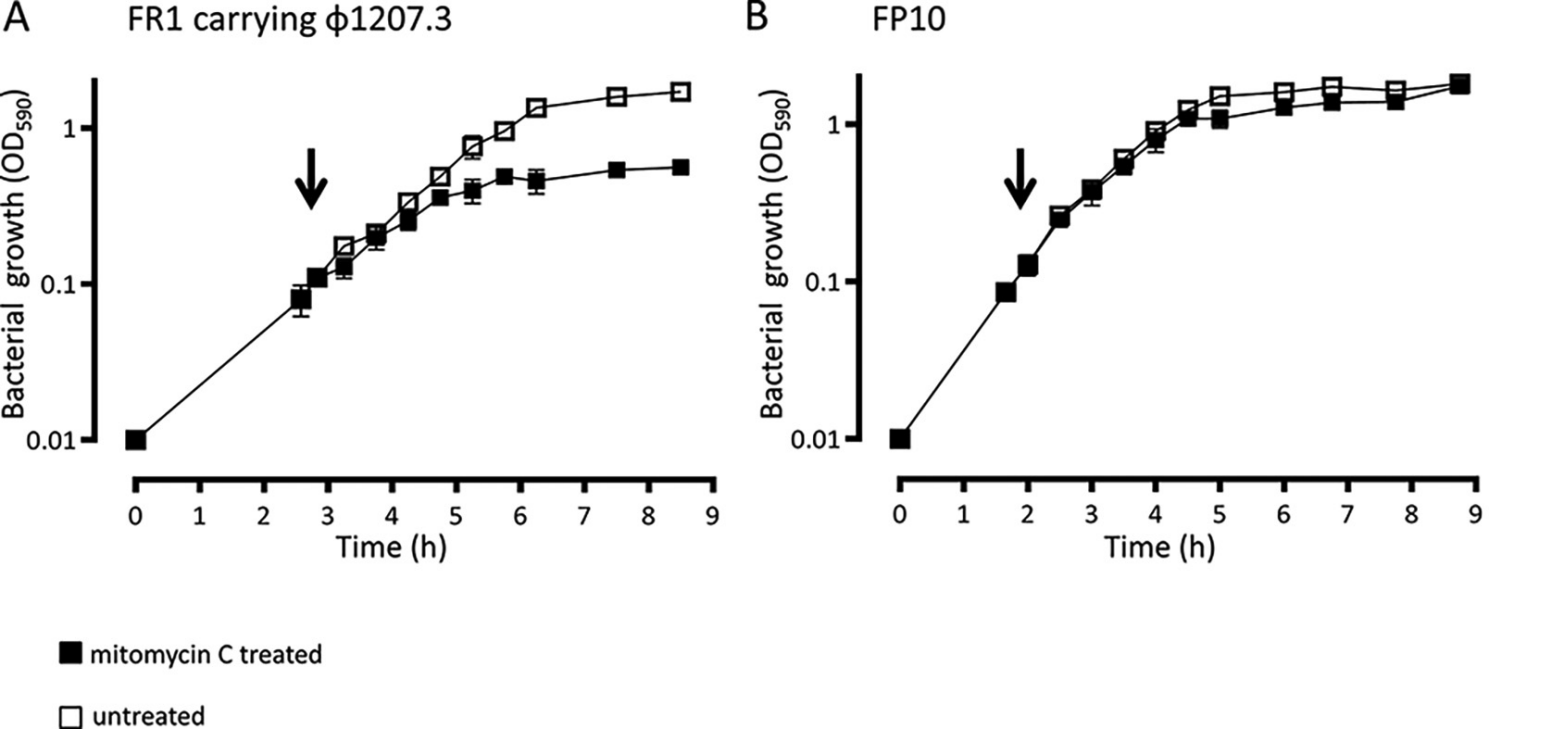
Φ1207.3 induction by mitomycin C. To determine if Φ1207.3 has a lytic phenotype, mitomycin C was added at a subinhibitory concentration (100 ng/mL) to liquid bacterial cultures in early exponential phase. (A) In the Φ1207.3-carrying strain FR1, mitomycin C causes a limited phage burst and a growth impairment, resulting in early entrance in the stationary phase (2.4 generations before of the untreated culture). (B) In the FP10 parental strain, mitomycin C produces only a slight growth impairment. The time point of mitomycin C addition is indicated by an arrow. Results are reported as means and standard deviations resulting from at least three independent experiments.

To assess if the Φ1207.3 enters the lytic cycle, liquid cultures of the Φ1207.3-carrying strain FR1 were induced with mitomycin C at subinhibitory concentration. Mitomycin C induced a limited phage burst and a growth impairment, resulting in early entrance in the stationary phase (Fig. 1). FR1 generation time was 51 min in the absence and 70 min in the presence of the mitomycin C stimulus. In the untreated cultures, the stationary phase was reached after 205 min, corresponding to 4 generations, whereas in the treated culture, it was reached after 121 min, corresponding to 1.6 generations. FP10 generation time was 54 min in the absence and 59 min in the presence of mitomycin C. In both the untreated and mitomycin C-treated cultures, the stationary phase was reached after 180 min, corresponding, respectively, to 3 and 3.3 generations. Hence, although bacteriophage Φ1207.3 does not seem to propagate efficiently by lytic cycle on plates, as it does not form lysis plaques, mitomycin C caused a significant, phage-specific growth impairment, as the treated culture reached the stationary phase 2.4 generations before the untreated culture. Almost no impairment of growth was observed in the isogenic progenitor control strain FP10 after mitomycin C treatment at a subinhibitory concentration (21).

### Detection of Φ1207.3 phage particles.

**FIG 2:**
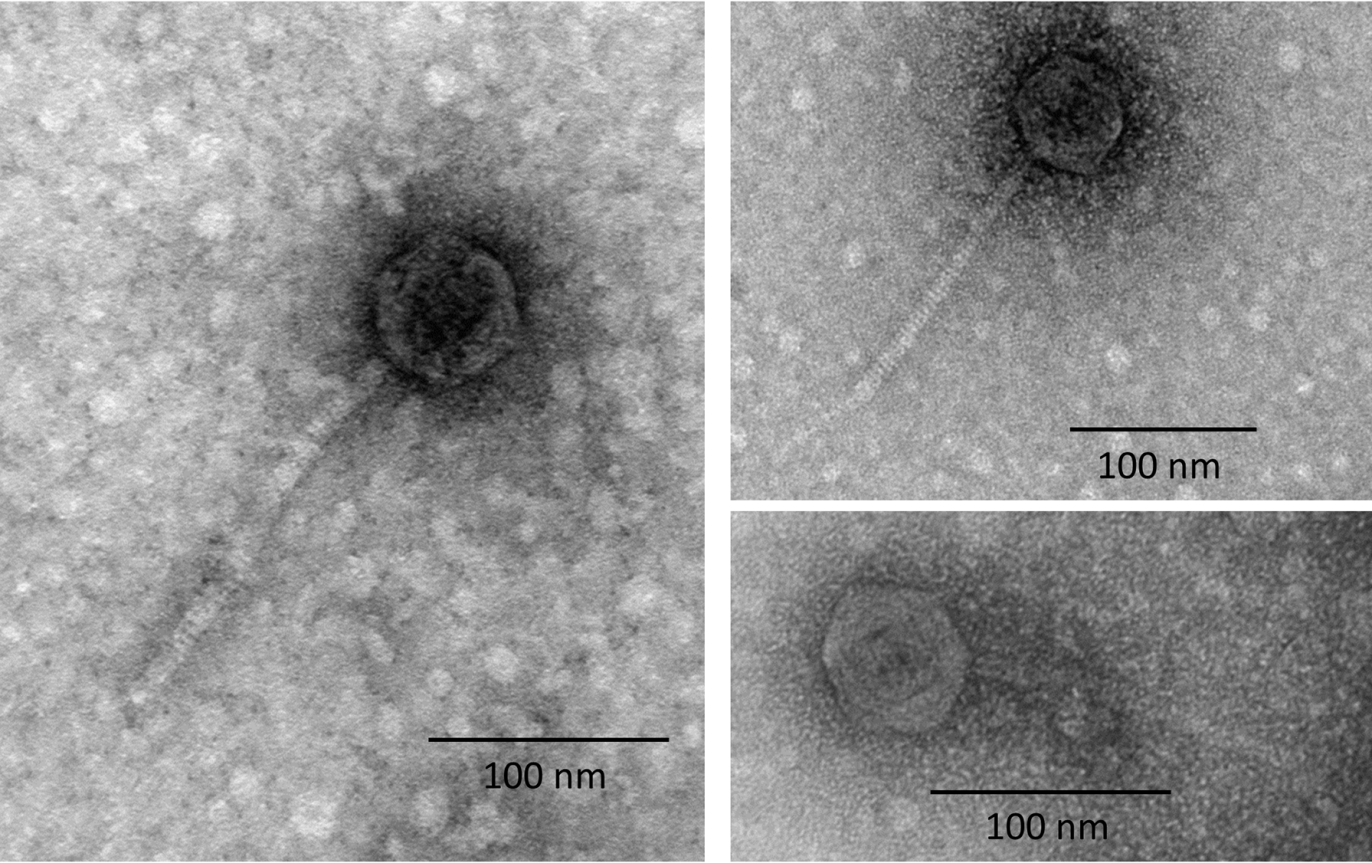
Transmission electron microscopy of the Φ1207.3 phage particles. TEM observation after negative staining of phage preparations shows the presence of phage particles with a morphology consistent with a siphovirus. The capsid shows an icosahedral symmetry and has a diameter of 62 nm, while the tail is noncontractile and 175 nm (±1 nm) long. The scale is reported for each panel.

In order to determine if Φ1207.3 is able to produce mature phage particles, we prepared supernatants from the Φ1207.3-carrying strain FR1. The supernatant recovered from a culture treated or not with mitomycin C was concentrated by sequential centrifugation and ultracentrifugation steps without filtering to minimize particle breakage. Observation of the ultracentrifuged supernatants at the transmission electron microscopy (TEM) showed the presence of phage particles with an icosahedral, electron-dense capsid and a long, noncontractile tail, with tail fibers visible in some images. The capsid was found to be 62 nm in diameter, while the tail was 175 nm (±1 nm) in length and 8 nm in diameter. The morphology observed is consistent with a siphovirus virion (Fig. 2). We specifically avoided a filtering step since it could significantly decrease phage recovery (22); moreover, we ultracentrifuged samples at a relatively low speed (20,000 × *g*) to preserve the integrity of viral particles (23). We conclude that Φ1207.3 is able to produce mature phage particles, suggesting it is not a defective prophage.

### Sequence analysis of Φ1207.3 predicted proteins.

To integrate the structural data from TEM, the head-neck-tail module search of Virfam was used to predict the Φ1207.3 phage morphology. Virfam analysis assigned Φ1207.3 to Siphoviridae with a cluster 2 type 1 neck, a family of phages that infect *Firmicutes* bacteria. Virfam analysis and a BLAST homologies search, conducted in public protein databases and the Pfam protein family database, predicted Orf38 to Orf47, Orf50, and Orf52 and Orf53 as putative structural proteins involved in the assembly of a complete phage particle (see Table S2 in the supplemental material). In addition, and as already observed, a predicted lysis module was found, composed of *orf53* and *orf54* coding for predicted holin and amidase proteins, respectively (Table S2) (12).

### Quantification of Φ1207.3 in enriched phage preparations.

Quantification of Φ1207.3 was performed by qPCR and with a semiquantitative approach based on the TEM imaging in phage preparations from both untreated and mitomycin C-treated FR1 cultures. Phage preparations, obtained after sequential centrifugation steps, were concentrated 308.57-fold compared to the original culture supernatant. We used qPCR to quantify the copy number of (i) joints between the ends of Φ1207.3 prophage sequence, i.e., the excised form of the Φ1207.3 genome, (ii) the macrolide resistance gene *mef*(A) carried by Φ1207.3, and (iii) the chromosomal reference gene *gyrB* in both phage preparations and bacterial cultures (Table 2). We estimated 1.03 × 10^7^ excised forms of Φ1207.3 genome per milliliter in the concentrated phage preparations obtained from the untreated cultures and 1.87 × 10^7^ excised forms per mL in the phage preparations from the mitomycin C-treated cultures. These values corresponded, respectively, to 3.34 × 10^4^ and 6.06 × 10^4^ excised forms per mL of the original culture supernatant. Within both phage preparations from untreated and mitomycin C-treated cultures, a 20- to 25-fold enrichment of phage genes compared to the bacterial *gyrB* gene was observed, proving that most of the phage signal does not come from contaminating bacterial DNA. Moreover, an enrichment in excised forms and *mef*(A) gene copy number was detected compared to the original bacterial culture. The excised forms were enriched 38-fold in phage preparations obtained from the untreated cultures compared to the bacterial culture, while the enrichment was 70-fold in the phage preparations obtained from mitomycin C-treated cultures. The *mef*(A) gene was enriched 7.8- and 38-fold, respectively. In contrast, a 10- and 5-fold decrease was observed for the chromosomal reference *gyrB* gene. To complement qPCR quantification, a semiquantitative approach based on the TEM was implemented to quantify the number of Φ1207.3 particles in the culture supernatant. We observed and counted phages in 30 meshes from at least 3 independent experiments. We estimated about 9.33 × 10^5^ ± 2.31 × 10^5^ phage particles per mL in concentrated phage preparations obtained from untreated cultures and 2.37 × 10^6^ ± 9.96 × 10^5^ phage particles per mL in the concentrated phage preparations from mitomycin C-treated cultures. These values corresponded, respectively, to 3.02 × 10^3^ and 7.68 × 10^3^ phage particles per mL of the original bacterial supernatant. The qPCR estimate was, in general, about 10 times higher than the TEM estimate. The discrepancy is possibly explained by the presence of free phage DNA in the preparation or, less likely, by the multiple genome copies packed in the phage head (24). However, in both cases, the fold increase upon mitomycin C treatment was similar. We conclude that mitomycin C, while having a clear and specific effect on the lysogen culture suggestive of prophage induction, resulted in a moderate production of viral particles (about a 2-fold increase compared to uninduced cultures), suggesting that the phage lytic cycle was somewhat impeded.

**TABLE 2:** PCR quantification of the Φ1207.3 phage genome

Sample^*a*^	Excised phages	*mef*(A)	*gyrB*
Bacterial culture	2.70 × 10^5^ ± 4.58 × 10^4^	4.87 × 10^6^ ± 3.34 × 10^6^	5.50 × 10^6^ ± 4.01 × 10^6^
Phage preparations	1.03 × 10^7^ ± 1.31 × 10^7^	3.81 × 10^7^ ± 4.05 × 10^7^	3.91 × 10^5^ ± 2.30 × 10^5^
Fold enrichment	38	7.8	0.1
Phage preparations (MitC^+^)	1.87 × 10^7^ ± 1.81 × 10^7^	1.86 × 10^8^ ± 2.13 × 10^8^	9.18 × 10^5^ ± 3.93 × 10^5^
Fold enrichment	70	38	0.2

a PCR starting templates were (i) the FR1 liquid culture, (ii) the phage preparations from the FR1 untreated culture, and (iii) the phage preparations from the FR1 mitomycin C-treated culture. Amplification target sequences were (i) the joints between the ends of Φ1207.3 sequence, i.e., the excised form of phage genome, (ii) the macrolide resistance gene *mef*(A) gene of Φ1207.3, and (iii) the chromosomal reference gene *gyrB*. Fold enrichment was calculated as the ratio between the value obtained from the phage preparation and from bacterial culture. Results are reported as means and standard deviations from 4 different experiments.

### Absence of plaque formation.

To further assess whether Φ1207.3 performs lytic cycles, phage preparations obtained from a mitomycin C-treated FR1 bacterial culture were used in plaque assay experiments. No plaques were observed with the indicator S. pneumoniae strain FP11. We conclude that, in our experimental conditions, a moderate cell lysis is taking place in liquid culture upon mitomycin C treatment (Fig. 1), but no plaque formation is detectable on plates of strain FP11. This is suggestive of a phage having a low burst size or a preference for growth in liquid cultures.

### Lysogenization with Φ1207.3.

**FIG 3:**
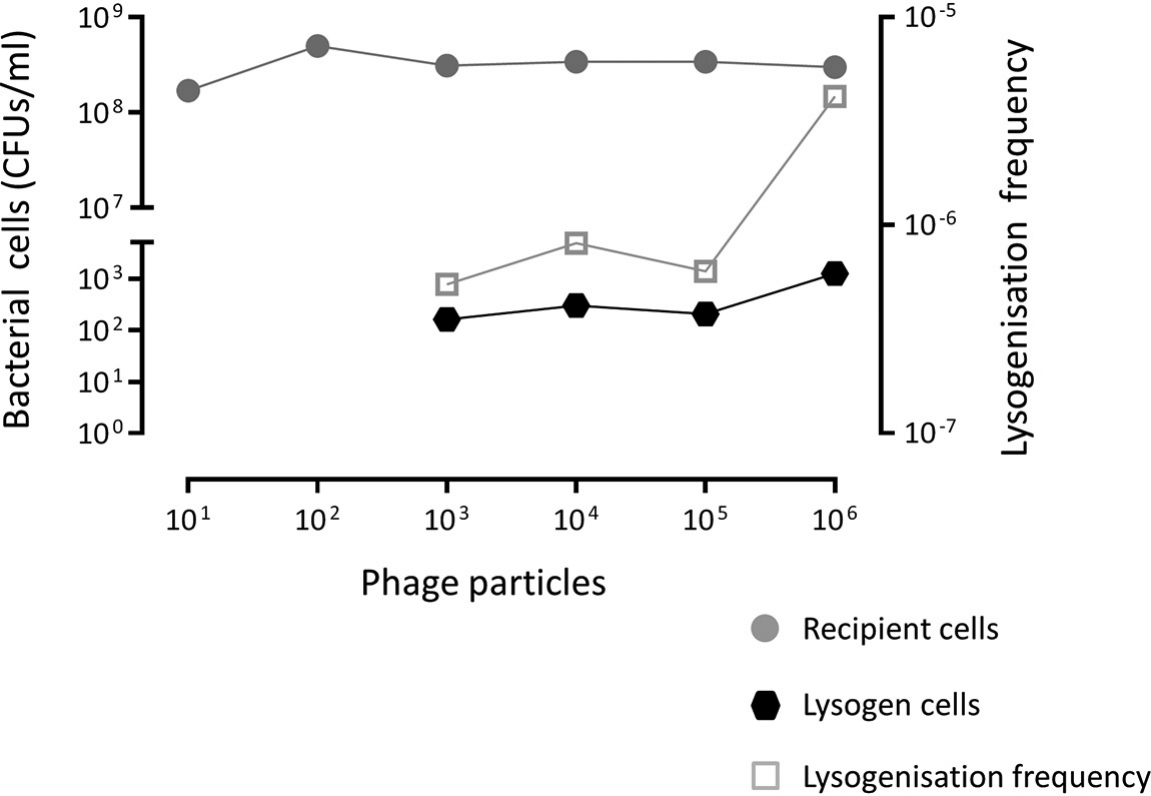
Lysogenization by Φ1207.3. Tenfold serially diluted phage preparations obtained from mitomycin C-treated FR1 culture were used to lysogenize the novobiocin-resistant and competence-deficient S. pneumoniae FP11. The minimum number of phages required to obtain a detectable lysogenization was 10^3^, whereas no lysogens could be detected (<2.8 × 10^−8^ lysogens per recipient) using lower phage numbers. The lysogenization frequency was calculated as number of lysogens per recipient cell.

**FIG 4:**
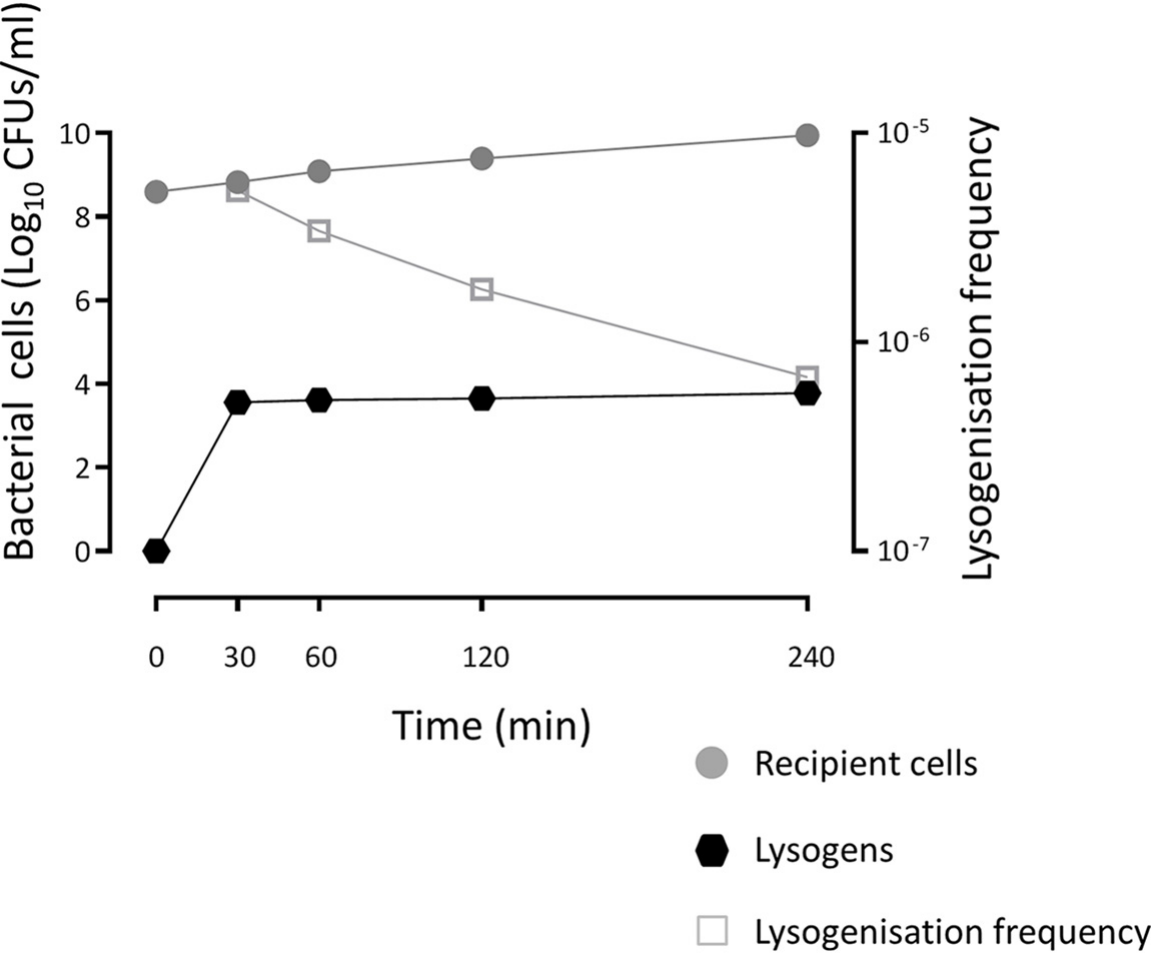
Kinetics of lysogenization by Φ1207.3. A time course lysogenization experiment shows that the number of recombinant lysogens is stable over time, indicating that lysogenization efficiently occurs within 30 min after contact of the phages with the recipient cells. The lysogenization frequency was calculated as the number of lysogens per recipient cell.

Finally, we tested if Φ1207.3 can infect and lysogenize S. pneumoniae FP11. For this purpose, we used concentrated phage preparations obtained from mitomycin C-treated culture and placed them into contact with the novobiocin-resistant and competence-deficient S. pneumoniae FP11 strain. Lysogens were obtained at an average frequency of 7.5 × 10^−6^ ± 5.7 × 10^−6^ per recipient, indicating that Φ1207.3 phage particles are able to lysogenize S. pneumoniae. The minimum number of phages required to obtain a detectable lysogenization was 10^3^, whereas no lysogens could be detected (<2.8 × 10^−8^ per recipient) using lower phage numbers (Fig. 3). Since we did not include a filtering step, our phage preparations also contained a residual number of bacteria (about 5 × 10^4^ CFU/mL), which were counterselected using novobiocin as a selection marker. As a further control, to rule out the contribution of whole bacterial cells to phage lysogenization (i.e., a conjugation-mediated transfer), we performed transfer experiments using 10-fold serial dilutions of an FR1 bacterial culture. When less than 6 × 10^6^ CFU of donor bacteria was used, no phage transfer was observed (<8.3 × 10^−9^ lysogens per recipient). Finally, we assessed the kinetics of phage lysogenization in a time course experiment, varying the lysogenization incubation time from 30 to 240 min (Fig. 4). The number of lysogens was stable over time, indicating that lysogenization efficiently occurs after 30 min from the contact of the phages with the recipient cells. On the other hand, the lysogenization frequency decreased over time as a consequence of increasing recipient CFU.

## DISCUSSION

Prophages play an important role in shaping the S. pyogenes genome, affecting the physiology and pathogenesis of this organism. Prophages are rarely found to directly encode antimicrobial resistance genes, and their transfer seldom occurs between different bacterial species (9, 25, 26). In S. pyogenes, the macrolide efflux genes *mef*(A)-*msr*(D) were found associated with several prophages, including Φ1207.3, Φ10394.4, Φ29862, Φ29961, Φ29854, and Φm46.1 and its variant, VP_00501.1 (15, 16, 18, 19). We found Φ1207.3 in the M1 serotype S. pyogenes clinical strain 2812A exhibiting the M phenotype of resistance to macrolides (10). Even if S. pyogenes strains devoid of phages were reported (27), most S. pyogenes genomes contain at least 2 prophages, with a maximum of 8 found in the genome of MGAS10394 (28). The genome of S. pyogenes 2812A contains other 2 prophages homologous to Φ315.3 and Φ5005.3 (our unpublished data). Since it is difficult to study the biology of a single prophage in the S. pyogenes original host due to its polylysogeny, Φ1207.3 was transferred to a prophage-free competence-deficient S. pneumoniae laboratory strain FP10. We demonstrated that Φ1207.3 is able to excise its genome, produce mature phage particles in S. pneumoniae with a morphology consistent with that of a siphovirus, and lysogenize sensitive recipients. In the S. pneumoniae lysogen hosting Φ1207.3, mitomycin C treatment does not induce a full lysis but only a limited growth arrest, and phage particle production is only 2-fold above the uninduced strain level. In a seminal work on pneumococcal bacteriophages, Bernheimer showed that mitomycin treatment at 100 ng/mL was necessary to recover phages from some strains, whereas other strains could yield phage also in untreated control cultures (29). In our hands, mitomycin C at a concentration of 100 ng/mL did not affect the growth of S. pneumoniae devoid of phage; in fact, the MIC was 250 ng/mL. Lysogenization by Φ1207.3 was obtained with concentrated phage preparations containing at least 10^3^ phage particles, while it could not be obtained using the nonconcentrated culture supernatant (10). Furthermore, lysogenization could only be detected when bacterial cells and phage particles were in close contact on an agar plate, suggesting that biofilm formation may be important for the transfer of this phage (30, 31). In conclusion, we demonstrated that Φ1207.3 is a partially functional phage with peculiar characteristics, i.e., (i) association with antibiotic resistance determinants, (ii) ability to lysogenize several S. pyogenes strains with different genetic backgrounds (our unpublished data) and other streptococcal species such as S. gordonii and S. pneumoniae (10), and (iii) the impairment in producing plaques on plates. Further investigations, e.g., in the original S. pyogenes host devoid of prophages (32), are required to fully elucidate the mechanism underlying the activities of this unusual bacteriophage capable of lysogenization while inefficient in its lytic cycle, as if upon infection, the choice between lysis and lysogeny was tilted heavily toward lysogeny.

## MATERIALS AND METHODS

### Bacterial strains and culture conditions.

Strains used in this work and their relevant properties are reported in Table 1. Streptococcal strains were grown in tryptic soy broth (TSB; BD) at 37°C. Starter cultures were sampled at an optical density at 590 nm (OD_590_) ranging from 0.2 to 0.3 and stored in 10% glycerol at −80°C. Solid media were obtained by supplementing TSB with 1.5% agar (BD) and 3% defibrinated horse blood (Liofilchem). When required, both liquid and solid media were supplemented with antibiotics at the following concentrations: 0.5 μg/mL erythromycin, 10 μg/mL novobiocin, 500 μg/mL streptomycin, 3 μg/mL chloramphenicol, and 500 μg/mL kanamycin (33).

### Transfer and lysogenization assays.

Transfer of bacteriophage Φ1207.3 from Streptococcus pyogenes to Streptococcus pneumoniae was obtained through plate mating experiments (20). Briefly, donor and recipient cells were grown separately at 37°C in TSB in the presence of the appropriate antibiotics. Upon reaching the end of the exponential phase (OD_590_ = 0.8), cells were mixed at a donor-recipient 1:10 ratio (i.e., 0.1 mL and 0.9 mL) and centrifuged at 3,000 × *g* for 15 min, the pellet was resuspended in 0.1 mL of TSB and plated on tryptic soy agar (TSA) plates supplemented with 5% blood. Plates were incubated at 37°C in the presence of 5% CO_2_ for up to 4 h, cells were then recovered with a cotton swab and resuspended in 1 mL of TSB supplemented with 10% glycerol and stored at −70°C. To select for recombinants, the mixture was then plated following a multilayer plating procedure as previously described (20, 33). Recombinant phenotypes were confirmed by genetic analysis on plates containing the appropriate antibiotics and genotypes by PCR analysis (34). To lysogenize pneumococcal recipients, a procedure similar to transfer assay was applied using phage preparation in place of the bacterial donor cells. For this, 0.9 mL of recipient cell culture (about 10^9^ CFU) was centrifuged, the supernatant was discarded, and the pellet was resuspended directly in 0.1 mL of the phage preparation (see below); the mixture was incubated for 15 min at 37°C before plating on TSA plates supplemented with 5% blood, incubating for 30 min at 37°C, and recovering cells with a cotton swab. Selection of bacterial lysogens was performed by multilayer plating in the presence of 1 μg/mL erythromycin. The number of phage particles used for the lysogenization assay was quantified by qPCR. To determine the minimum number of phage particles required for lysogenization, the phage preparations were 10-fold serially diluted.

### Mitomycin C induction and phage preparations.

Frozen starter pneumococcal culture was diluted 20-fold in 600 mL of TSB and grown at 37°C until reaching the early exponential phase (OD_590_ = 0.05 to 0.1), and then the culture was split into two aliquots, of which one was treated with 100 ng/mL mitomycin C (Sigma-Aldrich) (29, 35). After 2 h of incubation at 37°C, EDTA was added at a 10 mM final concentration, and the culture was centrifuged two times at 5,000 × *g* for 40 min at 4°C in 50-mL tubes to eliminate bacterial cells and cellular debris. The recovered supernatant of both induced and uninduced cultures was transferred into 6-polyallomer (36 mL each) centrifuge tubes (25 by 89 mm; Beckman Coulter) to which 1 mL of protease inhibitor cocktail (Sigma-Aldrich; catalog no. P8465) was added before ultracentrifugation at 20,000 × *g* for 2 h at 10°C in an Optima L-90K ultracentrifuge with the SW 32 Ti rotor (Beckman Coulter). Supernatants were carefully discarded by aspiration, and the 6 phage pellets were resuspended with 0.1 mL of TM buffer (50 mM Tris-HCl, 10 mM MgSO_4_) (29). A final volume of 0.7 mL was obtained by combining the 6 suspensions. The fold concentration of the enriched phage preparation was calculated as follows: 216 mL of supernatant/0.7 mL of suspension = 308.57×.

### Transmission electron microscopy.

Phage preparations were observed by TEM after negative staining by placing 3 μL of phage preparation onto a glow-discharged Formvar-coated 300-mesh copper grid. The phage preparation was allowed to adsorb for 2 min, excess liquid was removed with filter paper, and then a drop of 2% uranyl acetate was adsorbed on the grid and blotted dry. Samples were observed in a Tecnai G2 transmission electron microscope operated at 100 kV with a magnification of 60,000×. Measurements of head diameter and tail length were made to verify the homogeneity of the phage preparation. About 10 representative meshes were observed for each preparation, and 200 fields were observed in each mesh, covering the entire mesh. The number of phage particles for each grid was then calculated by multiplying the average number of phages in a mesh by the number of meshes in a grid (300). The number of phage particles per milliliter of phage preparation was then calculated assuming all phages of the 3 μL drop had adsorbed to the grid and was used to quantify the theoretical number of phage particles per milliliter of bacterial culture supernatant.

### Bioinformatic analysis.

The VIRFAM webserver (http://biaodev.cea.fr/virfam/) for remote homology detection of viral family proteins (36) was used to analyze the structural proteins of Φ1207.3 and assess the family of the head-tail-neck module. A BLAST search was conducted in public protein databases and the Pfam protein family database using default parameters, and only alignments with E values below 0.001 were considered.

### Quantitative PCR.

Quantitative real-time PCR assays were performed with the Kapa SYBR Fast qPCR master mix (2×) kit (Kapa Biosystems) on a LightCycler 1.5 (Roche) instrument, following the described procedure (37, 38). The reaction mixture contained, in a final volume of 20 μL, 1× Kapa SYBR Fast qPCR reactive, each primer at a 200-μM final concentration. The following templates were used: (i) the FR1 liquid culture, (ii) the phage preparations from the FR1 untreated culture, and (iii) the phage preparations from the FR1 mitomycin C-treated culture. Templates were incubated for 5 min at 90°C to allow bacterial cell and phage particle lysis, and then 1 μL was added to the PCR mix. The thermal cycling profile was as follows: 1 cycle of initial denaturation at 95°C for 3 min, 40 cycles of repeated denaturation for 0 s at 95°C, annealing for 20 s at 50°C, and extension for 10 s at 72°C. The temperature transition rate was 20°C/s in the denaturation and annealing steps and 5°C/s in the polymerization step. The melting curve was integrated at the end of the run by increasing the temperature from 40°C to 95°C with a ramping of 0.05°C/s and acquiring fluorescence continuously. The primer pairs used were (i) IF264/IF162, divergent primers, directed at the ends of the prophage genome, amplifying a fragment of 227 bp and used to quantify the excised phage; (ii) IF285/IF286, amplifying a 260-bp fragment, used for quantifying the *mef*(A) gene, and (iii) IF138/IF139, which amplified a 171-bp fragment of chromosomal *gyrB* gene, used for standardization of the results (see Table S1 in the supplemental material). Serial dilutions of chromosomal DNA with known concentration were used to build a standard curve for the *gyrB* gene by plotting the threshold cycle against the number of chromosome copies. This standard curve was recalled in the instrument software to standardize the number of *mef*(A) gene copies and excised phage genomes. Analysis of the melting curves was performed to discern desired amplification products from primer-dimer products. The number of excised phage genomes copies per milliliter of phage preparation was converted to the number of excised phage genomes copies per milliliter of bacterial supernatant. Fold enrichment was calculated as the ratio between the value obtained in the phage preparation and in the bacterial culture.

### Double agar overlay plaque assay.

In the plaque assay, 0.05 mL of the phage preparation (about 1.18 × 10^5^ phage particles, as quantified by TEM) were added to 0.1 mL of pneumococcal culture sampled in exponential phase (OD_590_ = 0.2 to 0.3) of the indicator strain, FP11. The phage-bacteria mixture was added to 3 mL of the top agar (TSB-0.4% SeaKem LE agarose), mixed gently, and poured into a 90-mm petri dish containing 25 mL of TSA supplemented with 10 mM CaCl_2_. The plates were dried for 10 min at room temperature and incubated at 37°C overnight in a 5% CO_2_ enriched atmosphere (39).
