# Supplementary material for: Streptococcus pyogenes Φ1207.3 Is a Temperate Bacteriophage Carrying the Macrolide Resistance Gene Pair mef(A)-msr(D) and Capable of Lysogenizing Different Streptococci
Source: Microbiol Spectr. 2023 Jan 10;11(1):e04211-22. doi: 10.1128/spectrum.04211-22 (PMC9927172; doi:10.1128/spectrum.04211-22)
Supplement: Supplemental file 1 — Tables S1 and S2. Download spectrum.04211-22-s0001.pdf, PDF file, 0.7 MB [file spectrum.04211-22-s0001.pdf]

Table S1. Oligonucleotide primers.

Name	Sequence (5’to 3’)	Target	Amplified product	GenBank ID: nucleotides
IF138	CAGATCAAGAAATCAAACTCCAA	Chromosomal *gyrB* gene, 5’ end	Chromosomal reference gene	CP079923.1: 713301-713323
IF139	CAGCATCATCTACAGAAACTC	Chromosomal *gyrB* gene, 3’ end		CP079923.1: 713451-713471
IF162	TGATGATTATATAAATTGTGAGTT	End of ϕ1207.3 genome, 5’ end	Φ1207.3 episomal form	AY657002.1: 52363- 52386
IF264	CTTGCTCTCACTTATTATATT	End of ϕ1207.3 genome , 3’ end		AY657002.1: 78-98
IF285	GGTCTTGTCTATGGCTTC	Macrolide efflux *mef*(A) gene, 5’ end	Phage reference gene	AY657002.1: 3402-3419
IF286	CTAAAAGTGGCGTAACCG	Macrolide efflux *mef*(A) gene, 3’ end		AY657002.1: 3644-3661

Table S2. ϕ1207.3 phage predicted structural proteins.

ORF (aa)	Predicted protein	Virfam homologous protein (identity) [E value/probability]	Pfam domains (aa) [E value]	Homologous protein ID / Origin (identity (%)) [E value]
orf38 (158)	Phage terminase, small subunit		Phage terminase, small subunit (50-146) [7.3e-31]	
orf39 (530)	Phage terminase, large subunit	D3 phage TermL (33%) [6e-77]	Phage Terminase (48-512) [8.9e-104]	
orf40 (413)	Phage portal protein	φ644-2 phage portal protein (39%) [100%]	Phage portal protein (32-381) [4e-121]	
*orf41 (*228)	Clp protease		Clp protease (13-177) [4.5e-40]	
orf42 (395)	Phage capsid protein		Phage capsid family (124-393) [3.7e-78]	
orf43 (67)	Phage adapter protein (Type 1)	Bcep176 phage Ad1 (24%) [98.46%]		
orf44 (109)	Phage head-closure protein (Type 1)	Φ105 phage Hc1 (25%) [99.96%]	Phage head-tail joining protein (7-104) [7.4e-15]	
orf45 (126)	Phage neck protein (Type 1)	Bv1 phage Ne1 (36%) [100%]		
orf46 (105)	Phage tail-completion protein (Type 1)	Bv1 phage Tc1 (41%) [100%]		
orf47 (196)	Major Tail Protein	bIL285 phage MTP (50%) [2e-48]		
orf50 (1039)	Tail tape measure protein		Phage-related minor tail protein (311-531) [2.1e-14]	AAG32164.1 / *Lactococcus* phage TP901-1 (152/693 (22%)) [1e-22]

ORF (aa)	Predicted protein	Virfam homologous protein (identity) [E value/probability]	Pfam domains (aa) [E value]	Homologous protein ID / Origin (identity (%)) [E value]
orf52 (967)	Host specificity protein		Prophage endopeptidase tail N-terminal domain (24-93) [1.8e-05]<br>Prophage endopeptidase tail (108-332) [5.4e-10]	AAK83249 /<br>*S. thermophilus* phage DT1.2 (113/418 (27%)) [5e-40]
orf53 (760)	Phage structural protein		Siphovirus protein of unknown function (DUF859) (1-512) [1.5e-214]<br>Holin family (638-753) [3.3e-40]	2FKK_A / *Escherichia* virus T4 (29/68 (43%)) [3e-06]
